# The Importance of Multidisciplinary Teams in the Early Detection and Management of Disseminated Nannizziopsis Infections in Solid Organ Transplant Recipients: A Case-Based Review

**DOI:** 10.7759/cureus.69920

**Published:** 2024-09-22

**Authors:** Augusta C Soyele, Sodjinin Kassa, Duha Zaffar

**Affiliations:** 1 Intensive Care Unit, University of Maryland Medical Center, Baltimore, USA; 2 Internal Medicine, University of Maryland Medical Center, Baltimore, USA

**Keywords:** brain metastases, end-stage renal disease (esrd), immuno-compromise, nannizziopsis obscura infection, rare fungi, travel history, treatment of brain metastases

## Abstract

This case report presents the clinical course of a 58-year-old female renal transplant patient with a complex medical history, including type 2 diabetes mellitus, hypertension, and chronic immunosuppression, who developed a disseminated *Nannizziopsis obscura* infection. The patient presented with persistent left leg pain and rapidly progressed to multisystem involvement, including central nervous system abscesses. Despite aggressive antifungal therapy and multidisciplinary management, her condition deteriorated, leading to a transition to palliative care. This report highlights the challenges in diagnosing and managing rare fungal infections in immunocompromised patients, highlighting the importance of early detection, advanced imaging, and a coordinated approach to care. The case also emphasizes the ethical considerations in balancing aggressive treatment with quality of life in terminal conditions.

## Introduction

Managing disseminated fungal infections in immunocompromised patients is a significant challenge, particularly in individuals with complex medical histories involving solid organ transplantation and chronic conditions such as type 2 diabetes mellitus, hypertension, and end-stage renal disease (ESRD). The emergence of rare fungal pathogens has further complicated this clinical landscape, necessitating heightened clinical suspicion, prompt diagnosis, and a multidisciplinary approach to treatment. This case report presents the intricate clinical course of a 58-year-old female with a history of type 2 diabetes mellitus, hypertension, ESRD status post-renal transplant, and multiple other comorbidities. Initially admitted with nausea and vomiting and worsening left leg pain, her condition rapidly escalated, leading to severe complications, including disseminated fungal infection, multiple abscesses, and eventual neurological decline.

The rare pathogen identified in this case, *Nannizziopsis obscura*, has been documented in only a few cases worldwide, primarily affecting immunocompromised individuals. The challenges of diagnosing and managing such rare fungal infections are well documented in the literature. For instance, Schilliger et al. (2023) highlighted the difficulty in recognizing and treating *Nannizziopsis* species due to their rarity and the lack of widespread clinical awareness [1]. Similarly, Miceli (2019) discussed the severe outcomes associated with central nervous system (CNS) involvement in fungal infections, emphasizing the need for timely imaging and early intervention to prevent irreversible neurological damage [2]. Pappas et al. (2010) further highlighted the vulnerability of transplant recipients to invasive fungal infections, stressing the importance of vigilant monitoring and individualized care in this high-risk population [3].

This case report contributes to the growing literature on rare fungal infections in immunocompromised hosts. It highlights the aggressive nature of such pathogens and the limitations of current therapeutic options. By exploring this patient’s clinical course, this report sheds light on the importance of early detection, aggressive management, and the consideration of palliative care when faced with overwhelming infection in the setting of organ failure.

## Case presentation

Patient X, a 58-year-old female with a significant medical history, including type 2 diabetes mellitus, hypertension, ESRD status post-renal transplant, anemia of chronic disease, adrenal insufficiency, chronic respiratory failure, and chronic immunosuppression, presented in October 2023 with Intermittent nausea and vomiting with persistent left leg pain for two weeks. The pain had worsened in three days, and she was unable to walk due to the pain. No fever or chills were present. She was taking an analgesic at home for the pain. She had been on immunosuppressive therapy as well, including tacrolimus, following her renal transplant, which placed her at an increased risk for opportunistic infections.

**Figure 1:**
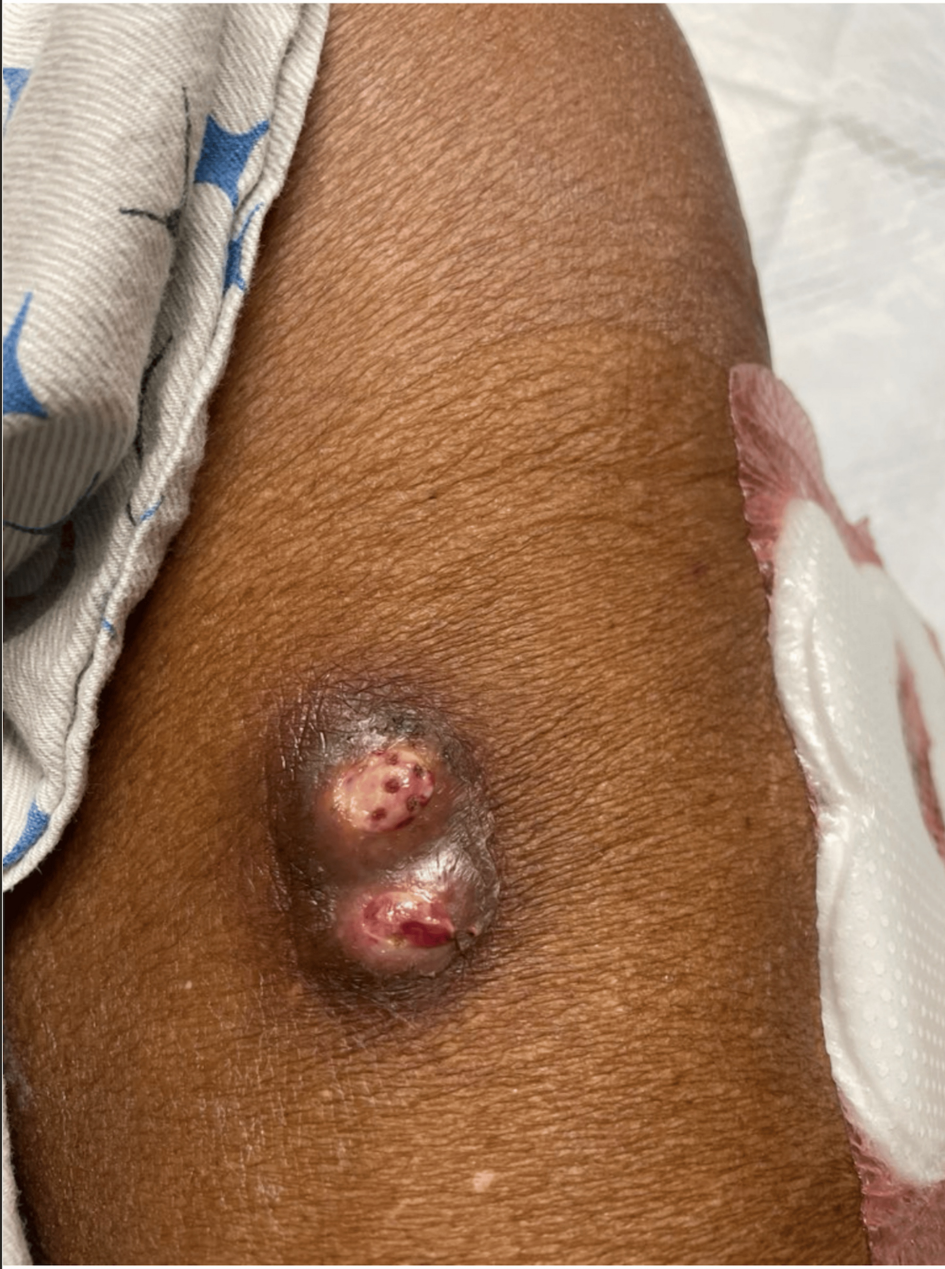
Left thigh: area of induration and fluctuation of the left lateral thigh with mucopurulent discharge.

Upon admission, a physical examination revealed a left leg pustular abscess and a left knee open abscess (Figure 1). The patient underwent incision and drainage of the left thigh abscess with copious amounts of purulent discharge. She was started on vancomycin and Zosyn, and wound culture was found to have hyphae. A CT scan of the left lower extremity showed a 5 × 3.5 × 3.2 cm multiloculated septated cystic lesion noted within the head of the lateral gastrocnemius muscle but no evidence of osteomyelitis. The patient was continued on Tylenol as needed for pain, vancomycin for broad-spectrum coverage, and Zosyn was discontinued due to acute renal injury. The patient was started on ceftriaxone with metronidazole for anaerobic coverage pending culture results and incision and drainage of the abscess. Based on a nephrology consult for organ transplant and immunosuppression management in a setting of worsening graft functions, a biopsy obtained in August 2023 showed no evidence of active T-cell-mediated allograft rejection; however, focal CD4 positivity history of antibody-mediated allograft rejection was observed with severe interstitial fibrosis and tubular atrophy, severe arterial sclerosis, and moderate arteriolar sclerosis/hyalinosis. Following a consult from infectious diseases, the patient had an area of induration and fluctuance in the left lateral thigh with mucopurulent discharge from the wound.

Additionally, an open wound inferior to the left knee had serosanguineous discharge but no limitation in range of motion. Upon review of her systems, the patient reported left leg pain. Given the cystic impression of a 1.5 × 3.5 × 3.2 cm multiloculated septated cystic lesion noted within the head of the lateral gastrocnemius muscle, the diagnosis of abscess was made, and a left lower extremity incision and drainage of gastrocnemius was collected which later showed hyphal elements. Given the risk of poorly controlled diabetes as well as immunosuppression, the patient continued posaconazole pending culture data. She was put on empirical treatment with daptomycin/ceftazidime/posaconazole. In early November, she noted the development of new nodules in the right hip and the right chest. She denied any trauma history and exposure to soil, dirt, or mold. She stated that the nodule appeared out of nowhere. The patient was born in Gambia but moved to the United States in 1988. She last visited Gambia two years ago. Upon physical examination, a 0.5-1 cm nodule on the right hip was non-tender and mobile. Incision and drainage results demonstrated yeast and amphotericin B was added to the medication regimen.

**Figure 2:**
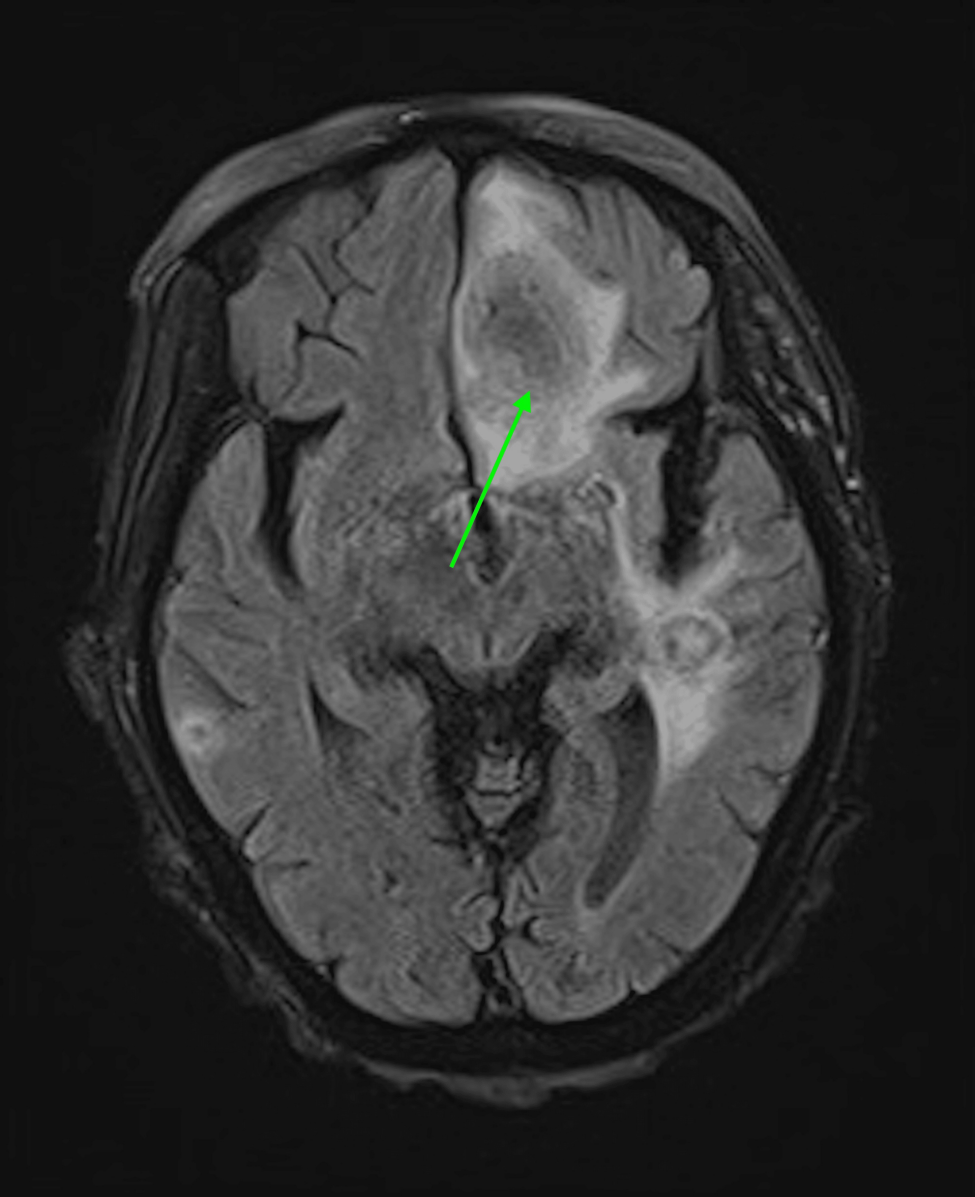
Initial brain MRI showing multiple ring-enhancing abscesses.

**Figure 3:**
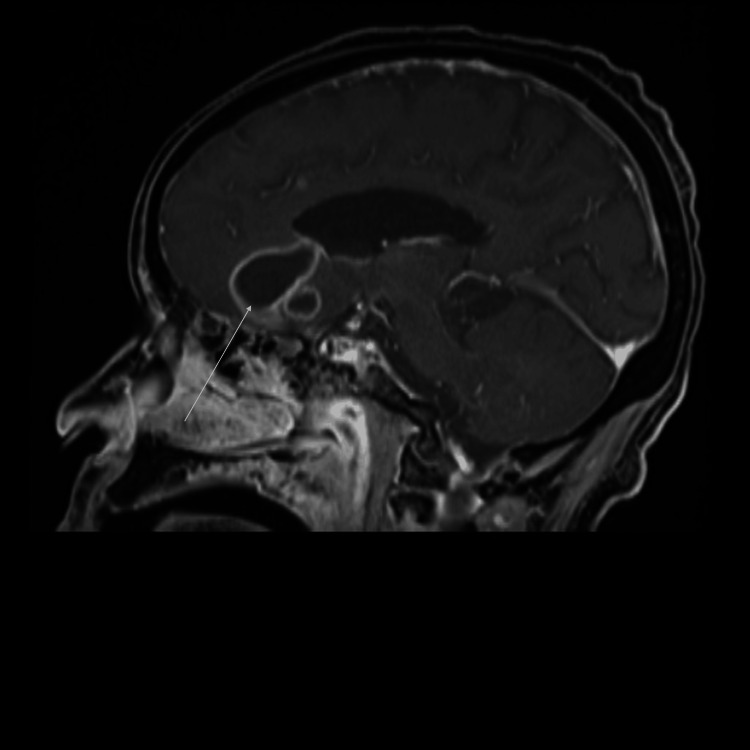
Initial brain MRI showing vasogenic edema in the inferior aspect.

Through gene sequencing, the mold was identified as *Nannizziopsis obscura*. This rare fungal infection has been documented in T-cell immunocompromised patients from West Africa. The most common clinical manifestations are subcutaneous tissue, lung nodules, and disseminated diseases. Given the nature of this infection, head CT was obtained given concern for CNS dissemination with this mold. CT showed multiple cystic lesions. The patient denied headaches or focal weakness. She denied any acute vision changes. Her CT showed multiple cystic lesions consistent with CNS brain abscesses. Brain MRI showed multiple ring-enhancing abscesses (Figure 2) with surrounding vasogenic edema in the inferior aspect (Figure 3) of the left frontal lobe and both temporal lobes with central restricted diffusion. She also had neck muscle abscesses. Posaconazole was changed to voriconazole for better CNS penetration. However, over the next few months, her condition deteriorated.

**Figure 4:**
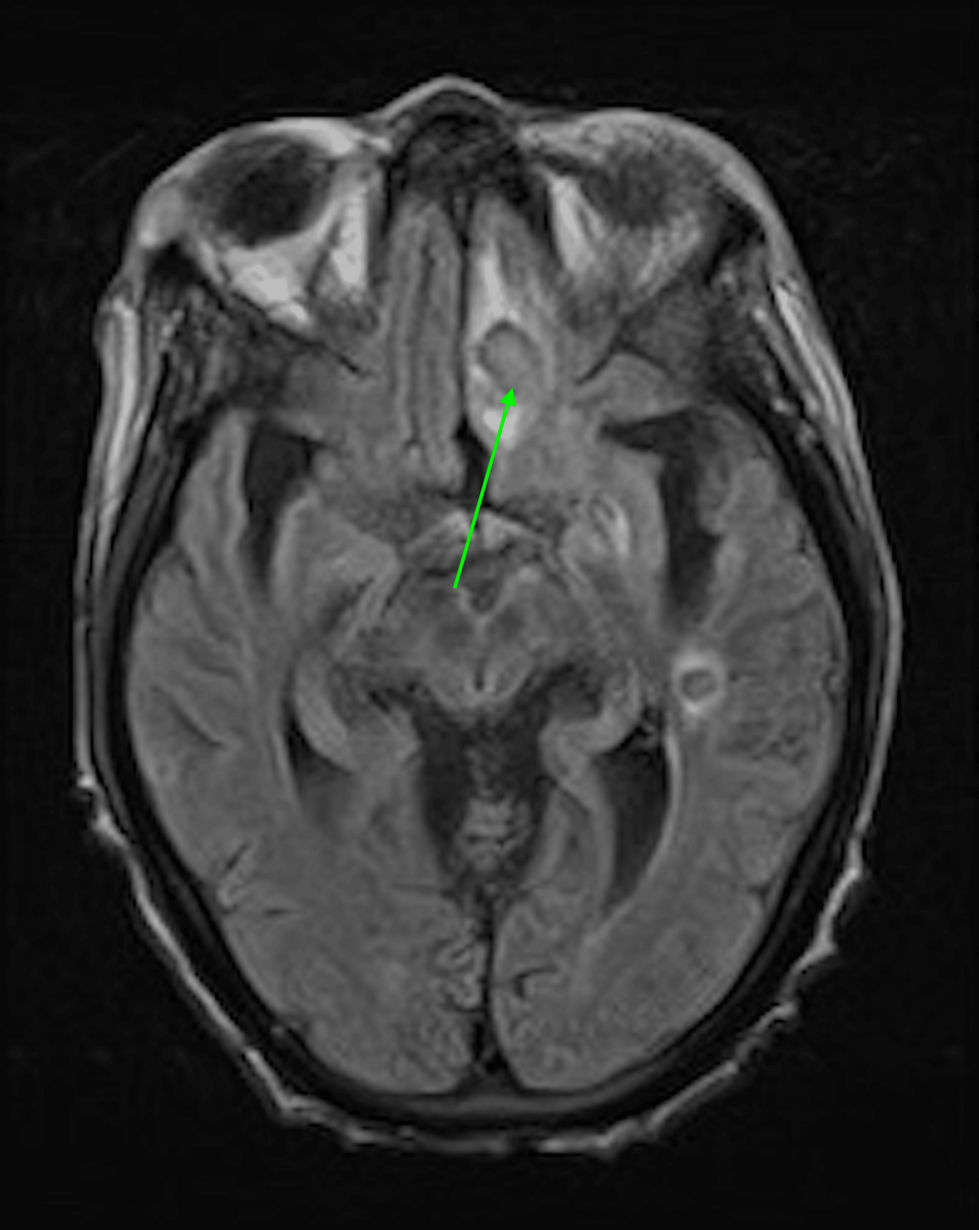
Follow-up MRI after the initiation of aggressive treatment.

**Figure 5:**
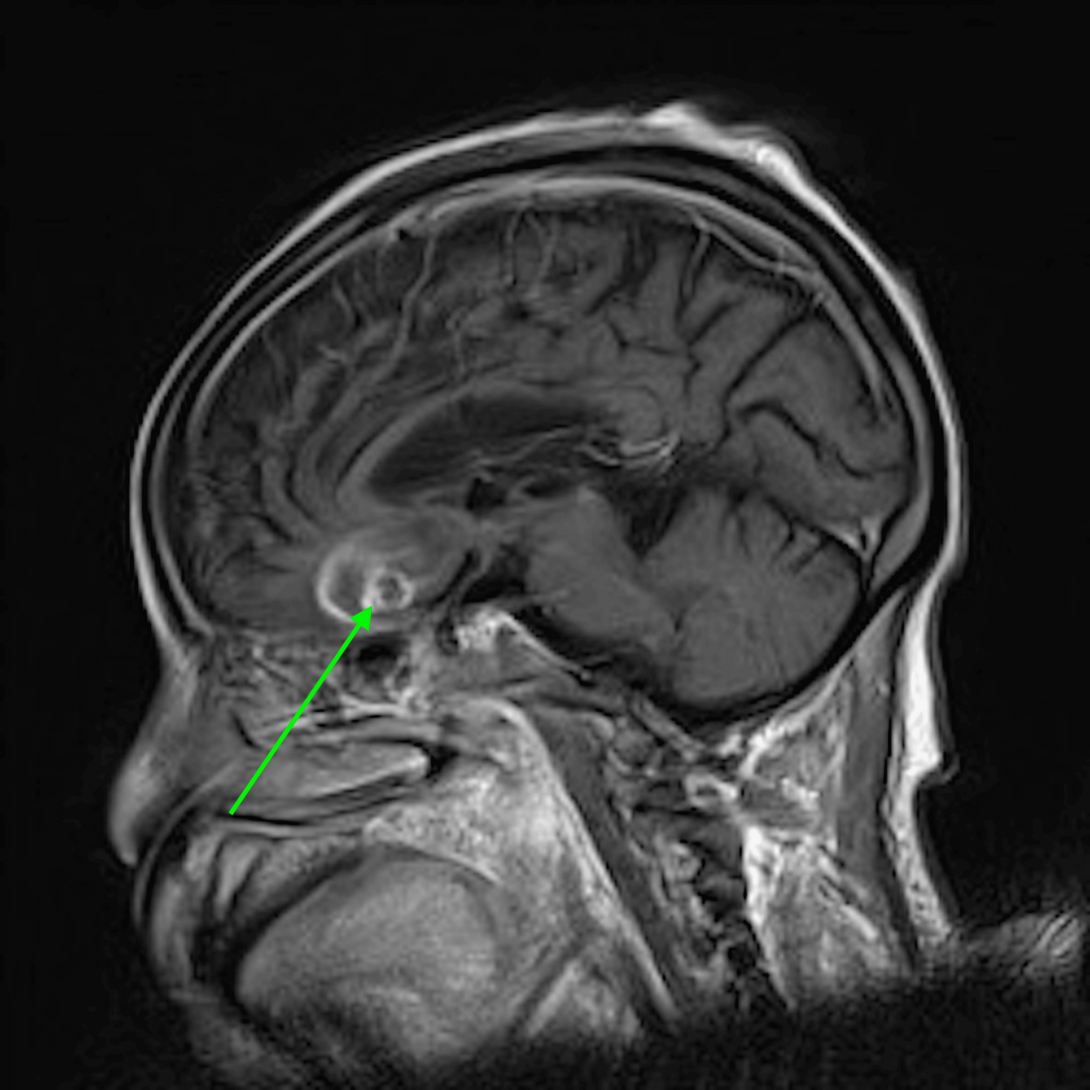
Follow-up MRI after the initiation of aggressive treatment showing multiple brain abscesses.

Given the rarity of *Nannizziopsis obscura* and its aggressive nature, an aggressive antifungal regimen was initiated, including amphotericin B and voriconazole. The choice of these agents was based on their broad-spectrum antifungal activity and the limited data available on the treatment of *Nannizziopsis obscura*. However, despite maximal medical therapy, her condition continued to worsen. Her neurological status declined, with an MRI (Figure 4) confirming multiple brain abscesses (Figure 5). The multidisciplinary team, including infectious disease, neurology, and transplant specialists, discussed the prognosis with her family.

Recognizing the poor prognosis and the progression of her disease despite aggressive treatment, the decision was made to transition the patient to palliative care. The decision process involved a careful consideration of the patient’s quality of life, the likelihood of therapeutic success, and the ethical implications of continuing aggressive treatment in the face of a terminal condition. She was made comfortable with symptom management, and the focus shifted to providing her with the best possible quality of life in her remaining time.

## Discussion

This case highlights the significant challenges in managing disseminated fungal infections in immunocompromised patients, particularly those with a complex medical history involving solid organ transplantation and diabetes. The case of patient X, diagnosed with a rare *Nannizziopsis obscura* infection, sheds light on the difficulty of early diagnosis and the critical need for timely intervention. *Nannizziopsis obscura* is an exceptionally rare pathogen documented in only a few cases worldwide, predominantly affecting immunocompromised individuals. Its dissemination, especially to the CNS, is associated with a high degree of morbidity and mortality, reflecting the pathogen’s aggressive nature and the limitations of current therapeutic options.

The patient’s clinical course mirrors broader challenges reported in the literature regarding managing rare fungal infections in transplant recipients. Delayed diagnosis, often due to the rarity of the pathogen and the non-specific presentation of symptoms, can lead to advanced disease by the time appropriate treatment is initiated. In this case, the progression of the disease despite aggressive antifungal therapy highlights the need for a high index of suspicion for opportunistic infections in immunocompromised patients. This is especially true for patients with a history of solid organ transplantation, where immunosuppressive therapy can exacerbate the risk of severe infections. The involvement of the CNS in disseminated fungal infections further complicates the clinical management of such cases. As seen in this case, the presence of brain abscesses posed significant challenges in diagnosis and treatment. The findings of Miceli (2019) parallel these observations, where CNS involvement in fungal infections such as aspergillosis necessitated early and aggressive imaging. The propensity of *Nannizziopsis obscura* to disseminate to the CNS highlights the importance of early detection through advanced imaging techniques and the need for prompt initiation of antifungal therapy to mitigate the risk of severe neurological complications.

In transplant recipients, the balance between effective immunosuppression to prevent organ rejection and the risk of opportunistic infections is delicate and fraught with challenges. The study by Pappas et al. (2010) emphasized the critical need for vigilance in this population, as rare pathogens such as *Nannizziopsis obscura* can lead to life-threatening infections. Patient X’s history of increasing tacrolimus doses, a common immunosuppressant, and the subsequent development of disseminated fungal infection emphasizes the complexity of managing such patients. The careful monitoring of immunosuppressive therapy and heightened awareness of rare infectious agents is crucial in minimizing the risk of severe infections in transplant recipients.

Comparisons with other case reports of disseminated fungal infections further elucidate the challenges faced in this case. For instance, the case report by Mohamad et al. (2013) described a renal transplant patient with disseminated mucormycosis, another severe fungal infection that required a multidisciplinary approach, including surgical intervention and antifungal therapy [4]. Similar to patient X’s case, the management of mucormycosis involved close monitoring of organ function and careful adjustment of immunosuppressive therapy. The case reports by Nourrisson et al. (2014) on *Nannizziopsis* species also highlighted the severe outcomes in patients with similar infections, pointing to the challenges in diagnosis and treatment due to the pathogen’s rarity and resistance to conventional therapies [5]. Unfortunately, despite aggressive treatment efforts, the patient’s condition continued to deteriorate, leading to the difficult decision to transition to palliative care. The poor prognosis associated with disseminated *Nannizziopsis obscura* infection, particularly in the context of immunosuppression and existing organ dysfunction, is consistent with the outcomes reported in other similar cases. The decision to focus on palliative care reflects the clinical realities of managing such rare and severe infections, where curative treatment options are often limited and the risks of further complications are high.

## Conclusions

The case of Patient X exemplifies the severe challenges posed by rare and aggressive pathogens in immunocompromised patients, particularly those with a complex medical history such as solid organ transplantation and diabetes. The progression of *Nannizziopsis obscura* infection, culminating in multisystem involvement and severe neurological complications, underlines the pathogen’s aggressive nature and the significant difficulties in managing such infections. Despite the implementation of maximal medical therapy, her condition deteriorated, highlighting the limitations of current therapeutic options and the often poor prognosis associated with such rare fungal infections. This case stresses the critical need for heightened awareness and early detection in similar clinical scenarios. The importance of individualized care plans, which may include the consideration of palliative care in end-stage disease, is paramount, as the balance between aggressive treatment and quality of life must be carefully weighed. In light of Patient X’s case, clinicians need to maintain a high index of suspicion for rare fungal infections in immunocompromised patients, especially those with a history of solid organ transplantation. The lessons learned from this case will contribute to a better understanding of the clinical course of such infections and the importance of personalized care in managing immunocompromised patients facing similar challenges. Further research into rare pathogens like *Nannizziopsis obscura* and developing more effective diagnostic and therapeutic strategies are urgently needed to improve outcomes in these high-risk populations.
